# Are We Teaching Nurses to Be Racist towards Aboriginal and Torres Strait Islander Peoples? A Critical Race Document Analysis of Discrete Aboriginal and Torres Strait Islander Health Courses

**DOI:** 10.3390/ijerph191811455

**Published:** 2022-09-12

**Authors:** Keera Laccos-Barrett, Angela Elisabeth Brown, Vicki Saunders, Katherine Lorraine Baldock, Roianne West

**Affiliations:** 1 UniSA Clinical and Health Sciences, University of South Australia, Adelaide, SA 5000, Australia; 2 College of Nursing and Health Sciences, Flinders University, Adelaide, SA 5042, Australia; 3 Central Queensland University, Cairns, QLD 4870, Australia; 4 Teaching Innovation Unit, University of South Australia, Adelaide, SA 5000, Australia; 5 Congress of Aboriginal and Torres Strait Islander Nurses and Midwives, Murarrie, QLD 4172, Australia; 6 Workforce Innovation, Charles Darwin University, Darwin, NT 0810, Australia

**Keywords:** Aboriginal, Australia, nursing, education, curriculum, racism

## Abstract

Background: Racism is responsible for health inequity and the harm perpetrated upon Aboriginal and Torres Strait Islander peoples by white institutions, building on attitudes and beliefs dominated by assumptions of white superiority. The National Aboriginal and Torres Strait Islander Health Curriculum Framework ‘Curriculum Framework’, released in 2014, was introduced to provide a framework for nursing programs and included the introduction of discrete Aboriginal and Torres Strait Islander health courses to draw attention to the relationship between racism health outcomes of Aboriginal and Torres Strait Islander peoples within health care settings. Methods: Using an Indigenist research paradigm with Colonial Critical Race Theory as the methodology and framework, this study presents a document analysis of discrete Aboriginal and Torres Strait Islander health courses taught in undergraduate nursing programs at 31 Australian Universities. Results: This work draws on the collective activism of Aboriginal and Torres Strait Islander nurses in challenging the systemic racism embedded in the Australian nursing curriculum. We demonstrate the utility of the Racial Segregation Audit Tool (RSAT), as an innovative approach to identify and respond to racism embedded in course learning outcomes. Conclusions: This study explores and uncovers how the learning outcomes assert the social construction of race as a tool of oppressive segregation.

## 1. Introduction

In 2020 more than 100 Indigenous and non-Indigenous Nurses and Midwives in Australia signed a call to action for Australian leadership in Nursing and Midwifery schools, colleges, and universities—to take racism seriously in teaching, learning and practice [1]. Geia et al. [1] acknowledge that despite development in curriculum reform initiatives, the depth of government policy in marginalising Aboriginal and Torres Strait Islander peoples is not taught; nor are evaluation process undertaken to identify improvement of cultural safety development because of education. The call to action speaks to the strength of the unity amongst Aboriginal and Torres Strait Islander nurses and their allies in nursing both nationally and internationally, especially in their commitment to addressing racism in the nursing profession. 

While the *National Aboriginal and Torres Strait Islander Health Plan 2021–2031* and the Australian Health Practitioner Regulation Agency (‘AHPRA’ [2]) call for a health system that is free from racism [3], First Nations scholars continue to highlight the need to counter whiteness norms and the deficit discourses that frame Aboriginal and Torres Strait Islander peoples within curriculum and to actively respond to explicit and implicit racism in nursing [1,4,5,6,7,8,9].

Whiteness is a form of dominance in a hierarchy of oppression officially declared in Australia through the declaration of *terra nullius* and the subsequent appropriation of Aboriginal and Torres Strait Islander sovereign lands in 1770 [10]. Racism itself is a multifaceted concept and social construct, and being racist is generally identified as holding beliefs and attitudes in the natural superiority of one’s own race over other races and the right to racial dominance [11]. Early Non-Indigenous researchers into the ongoing invasion of Aboriginal and Torres Strait Islander sovereign lands studied culture though racist/ethnocentric views and biases and created narratives of inferiority based on human stratification of race [12]. Butler [12] described racism within science as an ‘intellectual white-washing’ that has embedded racist norms within Australian society, perpetuated by Social Darwinism. Watego, Sing and Macoun [13] state that a society structured through and upon race is racist, echoing the works of Moreton-Robinson [10,14].

Racism in Australian health contexts is normalised, both institutionally and interpersonally [15]. Institutionally, racism is present through perceived cultural superiority of staff as impacting health policy, as well as the healthcare conditions and practices of staff [16,17,18,19] as evidenced by the work of Marrie and Bourke [20]. Interpersonally, Aboriginal and Torres Strait Islander peoples experience racism through health minimisation and misdiagnosis [15,21,22]. For instance, early diagnosis and treatment is significantly less likely for Aboriginal and Torres Strait Islander peoples compared to non-Indigenous Australians for numerous medical conditions [23]. This results from personal interactions with healthcare providers as well as interactions with systems developed by healthcare providers. 

Nurses comprise the largest proportion of health professionals likely to interact with people seeking care from the Australian healthcare system, including academic roles as well as educating student nurses [24]. Nurses, therefore, are a health profession with a critical role to play in dismantling the structures that support and create racist values within health education and facilitate racist and culturally unsafe care. However, Forsyth [25] found that segregation, assimilation policies and paternalistic ideologies were readily accepted by non-Indigenous Registered Nurses well into the 1970’s. Similarly, stories by Aboriginal and Torres Strait Islander nurses well into the 1990’s spoke of the deep embeddedness of racism within nursing as well as prejudice and resistance to change [26]. 

In 1991 Australia saw the *Royal Commission into Aboriginal Deaths in Custody* [27] endorse the *National Aboriginal and Torres Strait Islander Education Policy* [28] which called for the involvement of Aboriginal and Torres Strait Islander peoples in decision making processes around education. Similarly, the *Bringing them Home Report* [29] identified the impacts of a racist Australian legacy on education delivery and accessibility, urging Indigenous rights to self-determination and self-governing. Calls for anti-racist education reform have echoed in nursing spaces for decades [30]. Specifically, anti-racist education confronts racism, with the goal of eliminating and challenging its existence within the health workforce [13]. It requires individuals (and institutions) who are prepared to act against racism [31]. The activism of Aboriginal and Torres Strait Islander nurses led to the establishment of the Congress of Torres Strait Islander Nurses (CATSIN, later CATSINaM) [30,32]. The foundational reports spoke to the challenges faced by Aboriginal and Torres Strait Islander nurses in a system that promotes separation, hierarchy and power struggles, that is ‘the continuation of the assimilation process’ within nursing [30]. Within the reports were recommendations that all curricula relating to Indigenous health be guided and signed off by an advisory body of Aboriginal and Torres Strait Islander peoples [30,32].

Following the establishment of CATSIN, the *Gettin em and Keepin em* report [33] was released, calling for the involvement of Aboriginal and Torres Strait Islander peoples in the development and teaching of nursing education about Aboriginal and Torres Strait Islander peoples. Sherwood and Edwards [34] discuss institutional racism and the silencing of Aboriginal voices within health, and the urgent need to decolonise health professional workforce. However, Fredericks [35] notes that much of the training aimed at improving nurses’ knowledge and understanding of Aboriginal and Torres Strait Islander peoples at this time, was designed to raise awareness of ‘Indigenous issues’, rather than broaching anti-racism or at the very least valuing Aboriginal and Torres Strait Islander peoples. Despite the recommendations around anti-racism education and decolonised curriculum, the Australian Nursing and Midwifery Council (ANMAC) [36] *Registered Nurse Accreditation Standards* fell short of anti-racist curriculum expectations by only requiring “That the curriculum addresses specifically Aboriginal and Torres Strait Islander Peoples’ history, health and culture and incorporates the principles of cultural safety” [36]. 

The current *Registered Nurse Accreditation Standards* [37] have seen further developments including the requirement of the Registered Nurse program to have a discrete unit of study that is informed by the *Nursing and Midwifery Aboriginal and Torres Strait Islander Health Curriculum Framework* (‘N&M Curriculum Framework’ [38]), as well as the AHPRA *National Scheme’s Aboriginal and Torres Strait Islander Health and Cultural Safety Strategy 2020–2025* [2]. Yet, Drummond [39] observed that despite personal success in nursing education there is an overall marked devaluing of Indigenous ways of knowing and doing; that the very nature of academic institutions requires assimilation towards normalised whiteness, and that there is a divergence at the epistemological and ontological level of curriculum development [39]. 

Raising awareness of the embedded nature of racism in curriculum and identifying strategies to dismantle the teaching of racism, requires reviewing how racism is embedded and described in learning objectives. Learning objectives (also known as learning outcomes) form the basis of the course (unit of study), which in turn forms part of the Bachelor of Nursing program. Learning objectives describe the learning outcomes expected from the course, providing the basis of course content and determining all components of the course including student assessment [40]. To understand whether curriculum reinforces or dismantles racist ideologies among nursing students, requires evaluating the learning objectives of courses. To identify whether the strategies that are being implemented to target curriculum reform are translating into curriculum that does not oppressively segregate Aboriginal and Torres Strait Islander peoples, this study evaluates learning objectives in the discrete Aboriginal and Torres Strait Islander health courses in undergraduate nursing programs within Australian Universities. Our previous work [41] explored the development of a specific tool to identify and critique racism in curriculum. The current study builds on that research by using the RSAT tool to ask, *how do learning objectives within discrete Aboriginal and Torres Strait Islander peoples health courses in undergraduate nursing programs at Australian Universities assert the social construction of ‘race’ as a tool of oppressive segregation?*

## 2. Materials and Methods

### 2.1. Theoretical Background

The Indigenist research paradigm that frames this study aligns with the Indigenist research principles asserted by Professor Lester-Irabinna Rigney [42]. To assert my Aboriginality as a Ngarrindjeri woman in this research as a first author, I am required to maintain accountability to the communities to which I belong. Further details on how I have approached this are outlined in the previously published study protocol [41].

These Indigenist research principles are critical for how I situate myself within the research in culturally safe ways as well as the lens through which I view reality (ontology), my guiding morals and ethics (axiology) and the way I think of reality (epistemology), collectively [43,44,45,46]. The Indigenist research principles underpinning the research are: resistance as the emancipatory imperative, political integrity in research, and platforming our voices within research [42]. Such a positionality is a critical platform to support Indigenous ways of knowing and doing in research, where the very nature of the research is part of the racist oppressions that are our continued reality [42]. While Indigenous positionality within research could be considered bias by some disciplines, it is arguably necessary to respond to the ongoing need for assimilation into the non-Indigenous and white ways of knowing and conducting research, which is in itself an epistemological and ontological divergence from Indigenous ways of knowing and being [39]. 

Colonial Critical Race Theory (ColonialCrit) was chosen due to the difficulty in separating non-Indigenous research tools from the underlying beliefs which informed their creation [43]. Specifically, many positivist and post-positive paradigms do not align with the axiology of an Indigenist research methodology, yet ColonialCrit commits to challenging racism and oppression using political and resistance principles [42,43,47]. For instance, Critical Race Theory (CRT) originated in civil rights litigation in the US and where it is not modified for the context in which it is used it does not address placed-based histories of resistance struggle, emancipation and success such as Australian colonisation and racist government policies [48]. That is to say, the context of racism from which the theory of CRT was initially developed does not necessarily equate to the contexts of racism experienced by Aboriginal and Torres Strait Islander peoples in Australia [49], for instance, our knowledges, colonisation, as well as historical and contemporary policies. ColonialCrit adapts CRT to Australian contexts by centring Aboriginal and Torres Strait Islander peoples’ stories and histories in development of the theory [47]. ColonialCrit has four tenets;

- Recognising the social embeddedness of racism
- Asserting the social construction of race as a tool of oppressive segregation
- Privileging of stories and counter-storytelling
- Committing to social justice and praxis: incorporating activism.

The subsequent audit tool developed (RSAT) is informed by these tenets and draws on the works of Leonardo [50] and Gillborn [51] who explore whiteness theory and educational policy as an act of whiteness, respectively. Whilst whiteness theory is outside of the scope of this study these questions sit well alongside ColonialCrit in identifying power structures used to assert the social construction of race as a tool to oppressively segregate.

### 2.2. Development of the RSAT

The questions drawn from Leonardo [50] and Gillborn [51] have been used together to develop a Racial Segregation Audit Tool (RSAT) for the purposes of this study. Considerations for the analysis of the RSAT have been explored and published previously (Laccos-Barrett, Brown [41]. The RSAT is comprised of six questions which are provided in detail in Supplementary File S1; five questions provide a cumulative answer to question 6.

1. Who wrote the learning objective?
2. Is there avoidance of identifying with Aboriginal and Torres Strait Islander peoples and/or the racial experiences of Aboriginal and Torres Strait Islander peoples?
3. Who is placed as inferior and superior in the social construction of ‘race’ within the learning objective?
4. Is inequity explained by reference to any number of alternative factors rather than being attributable to a legacy of racism by those who belong to whiteness?
5. Who wins and who loses based on the priorities in the learning objective?
6. What will the likely effects of the learning objective be?

The questions within the Audit Tool have been selected and mapped across key documents [2,37,52,53,54] as shown in Supplementary File S1.

### 2.3. Validity of RSAT

An initial attempt to determine the face validity of the RAST tool was undertaken by three of the authors (KLB, AB, KB). Each of the authors independently used the RSAT tool to evaluate the same set of learning objectives, to explore levels of agreement across all RSAT items [55,56]. A Fleiss Kappa analysis showed a moderate level of agreement across all three authors at 0.760.

However, further considerations of analysis validity highlighted methodological limitations. Chilisa [57] cautions against the methodological dissonance between Indigenous and non-Indigenous research paradigms when undergoing a process of evaluation. This questions the degree to which a tool that assesses racial segregation and oppression can be validated by systems that assert white dominance through the failure to capture relational, political, discursive and historical powers in knowledge [10,58].

The results of the validity assessment were discussed by authors KLB, AB and KB, who identified a number of concerns regarding usability and clarity of the RSAT. Specifically, these concerns related to the disconnect between users who have an ontological and epistemological understanding of race struggles that are more closely aligned to whiteness and the impact this has on how they interact with the tool. This has resulted in considerations of user bias and the inability of the Fleiss Kappa analysis to fully determine the validity of the tool. The quantification of components of the tool, as well as a Racial Segregation Audit Tool (RSAT) user guide, has been developed as a means to attempt to reduce the bias of respondents who might otherwise ambiguously respond to the data as a grey area.

Validity within an Indigenist research paradigm requires the assessment of the cultural responsiveness of the evaluations [57]. Placing culture as the focal point within evaluation, in this instance, is determining whether the tool is representative of community and Indigenist research principles [42]. Ensuring validity for the RSAT has been an ongoing process of critical reflexivity throughout the development of the tool and the rationale behind it [41]. This is to ensure each component of the tool closely aligns with the aspirations of the Indigenist research paradigm: resistance and emancipation; political integrity and centring the voices of our activism within the nursing profession [42], together with the tenets of ColonialCrit [47]. This is interwoven throughout the design of the study and RSAT [41].

### 2.4. Data Inclusion/Exclusion Criteria

Data were included if they met the following inclusion criteria. Courses were offered by Australian Universities; had a specific focus on Aboriginal and Torres Strait Islander health; were included as discrete courses within undergraduate nursing programs; and were approved programs of study. Data were excluded if they were not offered by Australian Universities, were not approved programs of study, or were part of post-graduate programs, bridging or re-entry programs, or diplomas. Dual degrees such as BNursing/BMidwifery were assessed on a case by-case basis using the same criteria outlined above.

### 2.5. Data Collection

Data consisted of the course learning objectives of discrete Aboriginal and Torres Strait Islander health courses within Bachelor of Nursing degrees in Australia [41]. A list of programs of study approved by the Australian Health Practitioner Regulation Agency [59] was cross referenced with the 42 members of the Council of Deans of Nursing and Midwifery [60] to ensure all approved programs were identified. The learning objectives for data analysis were collected from the *course information pages* for discrete Aboriginal and Torres Strait Islander health courses, via their respective university websites. Data collection occurred in March of 2021.

Thirty-nine Australian universities were identified as offering approved programs of study for nursing. Three were excluded as they only offered postgraduate programs or diplomas. In total, thirty-six universities met the inclusion criteria. Of the thirty-six universities, five did not have their learning objectives publicly available at the time of study. For completeness of data, an application was made to the University of South Australia Human Ethics Research Committee (formally documented as exempt (application no: 203731)), to contact those universities who did not have their learning objectives available publicly. The Head of School (or equivalent) for the Nursing divisions were contacted via email on 10 February 2022, requesting a copy of the learning objectives for their Aboriginal and Torres Strait Islander health courses. There has been no acknowledgement or response from all 5 Head of Schools (or equivalent). 

### 2.6. Data Analysis

Thirty-one sets of course learning objectives from thirty-one different Australian universities were included in the analysis. Each of the 165 learning objectives were analysed using the RSAT questions. The results of this analysis were analysed quantitatively to determine to what extent racial segregation was within the learning outcomes. Additionally thematic analysis was used to provide a more nuanced analysis of question four, to explore how racial segregation was constructed within the learning outcomes [61]. 

## 3. Results

The descriptive statistical data were analysed in SPSS [62] and are presented in Table 1. In line with the RSAT tool located in the Supplementary File S1, the answers to questions one to five provided cumulative data to inform the answer to question six ‘what will the likely effects of the learning objective be?’. It is notable that in some instances learning objectives meet certain criteria but not others. For instance, where they may position Aboriginal and Torres Strait Islander knowledges as superior the same learning objective can also disempower and allude to power imbalances without acknowledging the legacy of racism by those belonging to whiteness. Examples used throughout the results section of this paper are shared for the purposes of the relevant question, rather than as an exemplar for all criteria. 

### 3.1. Question 1: Who Wrote the Learning Objective?

Question one serves to identify who leads the narratives of Aboriginal and Torres Strait Islander health. Authorship of Aboriginal and Torres Strait Islander courses and learning objectives are not publicly listed on university webpages in 100% (n = 165) of undergraduate nursing courses reviewed within the analysis. 

### 3.2. Question 2: Is there Avoidance of Identifying with Aboriginal and Torres Strait Islander Peoples and/or the Racial Experiences of Aboriginal and Torres Strait Islander Peoples?

Question two serves to identify whether whiteness is normalised within the learning objective, and whether there is an avoidance in identifying with the racial experiences of Aboriginal and Torres Strait Islander peoples. Whiteness uses othering as a tool to maintain power by separating those who are not within, or within proximity to whiteness as ‘the other’ [47,63]. Of the assessed learning objectives 47.9% (n = 79) avoided identifying with the racial experiences of Aboriginal and Torres Strait Islander peoples. An example of this avoidance is ‘discuss the legal, ethical, social and cultural issues that arise in the nursing care of Indigenous peoples across the lifespan’. Within this objective racially othering Aboriginal and Torres Strait Islander peoples creates a false dichotomy of ‘us and them’, in relation to nursing care rather than identifying the racialised barriers imposed by whiteness in nursing care [64]. In the example above, it is implied that the act of providing care for Aboriginal and Torres Strait Islander people is inherently problematic, where ‘legal, ethical, social, and cultural issues’ are seen as a natural outcome of care provision. This implies that the ‘problem’ itself is located with Aboriginal and Torres Strait Islander people, rather than the inherent racism of the health system itself. 

By contrast, 37% (n = 61) of learning objectives did identify with the experiences of Aboriginal and Torres Strait Islander peoples. For instance, ‘Examine Social and Emotional Wellbeing as defined by Indigenous health practitioners. The student will engage in Indigenous knowledges that underpin positive and holistic Social and Emotional Wellbeing for First Nations peoples. However, 15.2% (n = 25) of learning objectives were not applicable (N/A) as there was no identification of Aboriginal and Torres Strait Islander peoples or racialised experiences; for example, ‘demonstrating the knowledge and skills required to provide culturally safe nursing care’ or ‘Reflect on and apply professional standards, guidelines and codes of practice as they relate to culturally safe decision making and practice’.

Two institutions did not identify Aboriginal and Torres Strait Islander people within any of their Aboriginal and Torres Strait Islander course learning objectives.; only two objectives across the two institutions refer to ‘population groups’. A further two institutions did not acknowledge Aboriginal and Torres Strait Islander peoples as ‘people’. For example, ‘Discuss the health consequences of globalisation as it relates to population groups, health practices and health care service delivery, with particular reference to Indigenous populations’.

### 3.3. Who Is Placed as Inferior and Superior in the Social Construction of ‘Race’ within the Learning Objective?

Aboriginal and Torres Strait Islander peoples’ knowledges were situated either directly or indirectly as inferior to normative whiteness within 80% (n = 132) of learning objectives. This also applied to where the health professional is placed in a position of superiority within the learning objective itself. The learning objective ‘Apply the principles of cultural safety to enable culturally sensitive care to be applied across a range of populations and health care settings, but with particular reference to socially and culturally marginalized populations’ constructs ‘marginalised’ populations as inferior due to culture, whereas those not ‘culturally marginalised’ (normative whiteness) are constructed as superior. 15.2% (n = 25) of learning objectives were N/A where there is no identification of Aboriginal and Torres Strait Islander peoples or racialised experiences either directly or indirectly. Question 3 findings are consistent with the values of question 2. 

Aboriginal and Torres Strait Islander knowledges were placed in positions of superiority in only 4.8% (n = 8) of learning objectives that were analysed. Positioning of Aboriginal and Torres Strait Islander knowledge superiority in Aboriginal and Torres Strait Islander health contexts can be seen in the following learning objective:

*‘Have an understanding of self: by examining your values, prejudices, cultural beliefs and behaviours that affect the delivery of culturally safe nursing and healthcare for First Nations peoples. Students will identify their own potential prejudices, attitudes, beliefs, stereotypes and behaviours that could affect their nursing practice when caring for First Nation Australians.’*

### 3.4. Is Inequity Explained by Reference to Any Number of Alternative Factors rather Than Being Attributable to a Legacy of Racism by Those Who belong to Whiteness? 

Inequity was attributed to a variety of alternative factors other than racism in 25.5% (n = 42) of learning objectives, such as communication barriers or cultural issues. For instance, ‘Analyse determinants of health, cultural influences and models of health behaviour in relation to current health outcomes for, and the utilisation of health services by, Aboriginal and Torres Strait Islander peoples’. A further 29.1% (n = 48) of learning objectives attributed inequity to racism in a variety of ways, including colonisation and whiteness. A further 45.5% (n = 75) of learning objectives were N/A.

Thematic analysis was undertaken to determine the attributed reason for inequity within learning objectives that were categorised as ‘yes’ or ‘no’ (n = 90) and also includes one learning objective categorised as N/A. This particular learning objective indirectly identified racism as a cause of inequity whilst having previously not identified a racial group or experience: ‘examine the cultural self in the context of healthcare and racism through reflection’. The remaining learning objectives were marked as N/A and were not included in the thematic analysis outlined in Table 2. Of the learning objectives that were thematically analysed (n = 91), themes or recurrent ideas emerged from the data through an inductive analysis [65]. 

In thirty-five instances Aboriginal and Torres Strait Islander peoples were identified as the causal barriers to inequity in a variety of ways. This included being ‘culturally marginalised’, ‘cultural influences... on the utilisation of health services’, ‘engagement in health services’, ‘Indigenous healthcare problems’ and ‘contemporary implications of traditional aspects of life’. Of interest, is the number of times colonisation was alluded to by way of phrasing such as ‘historical events’ (n = 19), compared to the number of times colonisation is explicitly stated within the learning objective (n = 6). 

### 3.5. Question 5: Who Wins and Who Loses Based on the Priorities in the Learning Objective?

Question five explores whether the learning objective maintains and upholds notions of white superiority and dominance through the teaching of racist health professional behaviours such as paternalism, prejudice, low expectations and white saviourism. Alternatively, question five explores whether the learning objective acknowledges Aboriginal and Torres Strait Islander peoples’ sovereignty through narratives of self-determination, resistance and success [48]; whether Aboriginal and Torres Strait Islander peoples are positioned as the experts in Aboriginal and Torres Strait Islander health, and where students ‘see us on our terms’ [66]. 

40.6% (n = 67) of learning objectives maintain and uphold notions of white superiority and dominance. For instance, the following learning objective locates the source of inequality within social and cultural factors of ‘the other’ that are specific to Aboriginal and Torres Strait Islander peoples, and sit in contrast to implicit white superiority as the norm:

*‘Critically evaluate how social and cultural factors shape the health beliefs, experiences and outcomes of Aboriginal and Torres Strait Islanders and other cultural groups’*

The majority of learning objectives (56.4%, n = 93) construct narratives of false neutrality in which structural factors are acknowledged without identifying whiteness or racism as the primary drivers of inequality. This can be seen in the following learning outcome, where there is an acknowledgemt of historical and current determinants of health without specific acknowledgement of racism as a fundamental driver of those determinants:

*‘Describe the context of health and wellbeing for Aboriginal and Torres Strait Islander peoples, including historical and current determinants of health, human rights, principles of social justice, transpersonal caring, primary health care needs utilising strength-based approaches, and policy considerations’*

Only 3% (n = 5) of learning objectives acknowledge Aboriginal and Torres Strait Islander peoples’ sovereignty, as evident in learning objectives such as ‘be able to outline Aboriginal and Torres Strait Islander ways of knowing, the importance of sovereignty and self-determination, and cultural protocols’. 

### 3.6. Question 6: What will the likely Effects of the Learning Objective Be? 

Question 6 explores the cumulative answers to question one to five in determining whether the learning objective is working towards anti-racist curriculum; or, whether the learning objective asserts or maintains the social construction of race as a tool of oppressive segregation. 

Of the 165 learning objectives assessed, 85% (n = 135) assert or maintain oppressive segregation of Aboriginal and Torres Strait Islander peoples. For instance, ‘explore practical and theoretical issues underpinning work with Aboriginal and Torres Strait Islander people and communities including interpersonal communications skills’.

A further 5.5% (n = 9) of learning objectives work towards anti-racist curriculum, for instance, ‘be able to outline Aboriginal and Torres Strait Islander ways of knowing, the importance of sovereignty and self-determination, and cultural protocols’. Another 11.5% (n = 19) are N/A, as determined by not applicable answers to questions two to five; this included learning objectives such as ‘demonstrate understanding of principles of community engagement’. 

## 4. Discussion

In this study, the RSAT was used as an innovative approach to determine how learning objectives within discrete Aboriginal and Torres Strait Islander peoples health courses in undergraduate nursing programs at Australian Universities assert the social construction of ‘race’ as a tool of oppressive segregation. Data analysis reveals a disparity between anti-racist teachings and learning objectives that continue to assert race as a tool of oppressive segregation. Specifically, the key finding highlights a divergence from the plans of creating a health system ‘free from racism’ [3] and the strategies [2,67] and frameworks [38,40] which inform nursing curriculum and the way in which they are implemented within discrete Aboriginal and Torres Strait Islander peoples health courses. 

Findings from this study highlight that, even where there are detailed curriculum frameworks and resources available [38,40], further steps need to be taken to reduce the perpetuation of racism within curriculum. Since the release of the *Curriculum Framework* [40], further calls for Aboriginal and Torres Strait Islander curriculum reform have explicitly targeted racism [1,4,5,6,7,8,9]. Many learning objectives were direct excerpts from currently available curriculum frameworks [38,40]; however, our analysis found that these excerpts can still lend support towards institutional passivity regarding anti-racism. As such, a key recommendation from this study is that the currently available curriculum frameworks require a comprehensive review, to ensure that learning outcomes, content and pedagogical approaches are actively challenging and dismantling racism. In a 2012 audit of pre-service teacher training Moreton-Robinson et al. [68] found that ‘Unless the relationship between racial privilege and racial disadvantage is understood the development of an effective Indigenous pedagogy remains beyond the scope of the national standards’. However, culturally responsive pedagogies, for instance, provide frameworks towards curriculum that are derived from critical theory, centring social justice and the classroom as a tool for social change [69,70,71,72,73]. Similarly, Ramsden [74] discusses anti-racism in nursing education as a foundational concept for the application of cultural safety education. Exploring pedagogical approaches with a focus on ‘race’ may assist in developing Aboriginal and Torres Strait Islander nursing curricula that works towards a health system ‘free from racism’ [3]. Nevertheless, there are factors to consider which require further exploration outside of the scope of this study; for instance, a recent literature review of anti-racist efforts in nursing education in the United States and Canada highlights concerns around the ability and literacy of nurse educators to deliver anti-racist, anti-discriminatory, post-colonial and intersectional perspectives [75]. 

In addition to the main findings, this study also highlights where institutional passiveness lends support towards maintaining racism within nursing curriculum. This includes authorship and transparency issues, as well as the ability of institutions to achieve course accreditation while producing learning objectives that dehumanise, problematise and marginalise Aboriginal and Torres Strait Islander peoples through narratives of white superiority, fostering education on paternalism and white saviourism. Five tertiary institutions have not listed their course learning objectives online, and upon request for inclusion in this study there was no acknowledgement of communication. Macoun, Parker [76] talk of the ongoing pattens of colonial violence through the normalisation of coloniser sovereignty and false neutrality of ignorance. This is a far contrast to the *Indigenous Strategy 2022–25* [67] focus area ‘racism and cultural safety’ and Universities Australia’s commitments on anti-racism. Explorations of Australian universities continue to highlight the false neutrality of ignorance which upholds and maintains the violence perpetuated in narratives built by and for colonialism [77,78,79]. White ignorance and white innocence are employed under the guise of a progressive façade, actively refusing to engage in processes to dismantle racist systems and maintaining systems of racialised violence [79]. 

Accountability is explored further in Question 1 of the RSAT, ‘who wrote the learning objective?’. This question identifies institutional transparency of authorship, who drives the narrative of Aboriginal and Torres Strait Islander peoples in the development of the learning objectives. Authorship is not explicitly stated across 100% of courses in the study. While this appears to be a general approach across most courses in the programs, the findings talk to the normalisation of power hierarchies, lack of clarity, systemic racism, and a lack of accountability across universities. This also talks to the history of academic institutions silencing the voices of Aboriginal and Torres Strait Islander peoples [34,80,81]. Moreton-Robinson [78] states that the silencing of Aboriginal and Torres Strait Islander voices is how racism operates in knowledge production, through ‘white ignorance’, as a process to invalidate, ignore and silence Aboriginal and Torres Strait Islander voices. ANMAC [37] have attempted to incorporate a process to challenge this, requiring the education provider to undertake consultation in the design and management of the program from external representatives including Aboriginal and/or Torres Strait Islander peoples, such as advisory committees [82]. However, the information of who drives the narrative of Aboriginal and Torres Strait Islander peoples is held by the institutions and the accreditation bodies. As discussed, institutional racism is present in the silencing of Aboriginal voices. Current recommendations on transparency and accountability of *the Higher Education Standards Framework (Threshold Standards) 2021* [83] require universities to ‘support participation of’ and to be ‘sensitive to Aboriginal and Torres Strait Islander knowledges and cultures’, but there are no requirements for accountability or transparency at the legislative level. While Universities Australia (UA) [67] have explicitly committed to accountability regarding the use of Aboriginal and Torres Strait Islander knowledge and cultures in universities, there is no specification for transparency. Frameworks and processes for developing an institutional accountability and transparency model could be readily adapted from current research ethical guidelines [84,85,86]. 

The findings of the analysis indicate that nearly half of learning objectives avoided identifying with the racial experiences of Aboriginal peoples (Q2); specifically Aboriginal and Torres Strait Islander peoples are positioned as ‘other’, separate to whiteness. Two institutions referred to ‘population groups’ whilst not acknowledging Aboriginal and Torres Strait Islander peoples, and a further two institutions failed to acknowledge personhood. Watson describes Australia’s history of colonialism as a denial of Aboriginal existence [87]. However, within some of the learning objectives assessed there are still components of dehumanisation, as well as iterations of the ‘Aboriginal problem’ [87]. This supports Zimmerman et al. [88], where it was identified that it is possible to achieve accreditation with curriculum that is not aligned to the *Aboriginal and Torres Strait Islander Health Curriculum Framework* [40], but further suggested that accreditation is achievable with curriculum which advances narratives of white superiority and promotes racism. In fact, Aboriginal and Torres Strait Islander knowledges were either directly or indirectly positioned as inferior to normalised whiteness in 80% of learning objectives (Q3). Such racialisation of Aboriginal and Torres Strait Islander knowledges presents a deficit discourse, to maintain a narrative of white superiority [89]. Fogarty et al. [90] presents strengths-based approaches as an alternative to deficit discourse which is grounded in Indigenous ways of knowing and identifies numerous strengths-based approaches to do such. 

Use of the RSAT further identified that numerous learning outcomes attribute inequity to factors that lay blame with Aboriginal and Torres Strait Islander peoples as ‘other’ (Q4). Specifically, this included problematising Aboriginal and Torres Strait Islander peoples as ‘culturally marginalised’ with ‘cultural issues’ and ‘cultural influences’ impacting poor service engagement. These present patterns of deficit discourse, that is disempowering patterns of thought, language and practice that can homogenise and dehumanise Aboriginal and Torres Strait Islander peoples [89]. From the thematic analysis itself there appears to be a tendency to shift away from words such as ‘colonisation’, ‘whiteness’ and ‘racism’ in favour of phrasing such as ‘historical events’. The avoidance of wording such as ‘racism’ is interesting given that the learning objectives assessed were of discrete Aboriginal and Torres Strait Islander health courses. A broader review of racism in nursing curriculum is suggested. 

Findings from the data analysis identified a strong tendency of disempowerment or ‘neutrality’ (Q5), with only 5.5% of learning objectives driving narratives of sovereignty, self-determination, resistance, and success (Q6). The Australian Health Practitioner Regulation Agency [2] states that culturally safe practice is free from racism and is determined by Aboriginal and Torres Strait Islander peoples; and the *National Aboriginal and Torres Strait Islander Health Plan 2021–2031* aims to identify and eliminate racism (DoH [3]). However, it can be questioned the degree to which nursing curriculum is working towards a health system that is ‘free from racism’ if only four learning objectives across 31 universities explicitly identify racism and if narratives do not foreground sovereignty where curriculum ‘about us sees us on our terms’ [66]. Watego, Singh and Macoun [13] discuss that anti-racism should confront racism, and that individuals and institutions both must be prepared to act against racism [31]. In the context of Australian undergraduate Nursing curriculum and topics which are specific to Aboriginal and Torres Strait Islander health, currently anti-racist curriculum is not the norm. In fact, what is evident following the analysis is the significant finding that curriculum currently perpetuates rather than challenges racism. 

### Limitations and Strengths

Racism in itself is not static, it is always evolving, changing and adapting where methods to oppress and segregate adapt in order not to challenge the racialised hierarchies underpinning society [51]. In this way it is important to note the limitations of using a quantitative tool that does not identify racism in all of its nuances, or account for the lens of the person who is interpreting the data and the way they perceive and interact with the data. The nature of racism, as always adapting and evolving as a means to maintain white dominance, ensures that quantitative tools are outdated quickly [13]. The RSAT does not give a total overview of racism within the learning objectives discussed, rather focuses on oppressive segregation. The analysis only tells part of a story and needs to be viewed in such light with a critical lens. It is imperative that educators who use the tool ensure that the auditing process is done from a place of criticality and openness. 

A key strength in the analysis is the use of a consistent framework for all learning objectives [38], allowing a modicum of curriculum standardisation for comparison. Specifically, the research reflects the adoption of the *N&M Curriculum Framework* [38] into the ANMAC *Registered Nurse Accreditation Standards* [37]. A further key strength to this study was the framework provided by the ColonialCrit tenets [47] as well as the foundation of the Indigenist research methodology [41] to guide a research agenda that does not centre white ways of knowing, instead centralising emancipation from white racial oppression. 

The findings from the study highlight a divergence from the aspirations of the *National Aboriginal and Torres Strait Islander Health Plan 2021–2031* [3] to have a health system free from racism. Since the release of the 2014 *Aboriginal and Torres Strait Islander Health Curriculum Framework* [40], national priorities for health are more orientated towards addressing racism [2,3]. The RSAT offers an innovative tool for critically analysing the narratives that underpin nursing curriculum in Australia and identifying where and how racism is reproduced within Aboriginal and Torres Strait Islander health courses. A key challenge will be the institutional tendency to engage in ‘neutrality of ignorance’ to maintain current power hierarchies of white dominance. Further research into an anti-racist curriculum framework that accounts for the nature and embeddedness of racism would be welcomed.

## 5. Conclusions

The impact of efforts in Australian Universities to respond to racism in the health professions through education are poorly monitored and evaluated. Additionally, implementation of anti-racist learning aspirations and objectives have been varied. Changes are being made with strategies, plans and frameworks to inform and shape curriculum towards the AHPRA aspiration for a health system ‘free from racism’. However, through the development and application of the RSAT we have found that educational approaches to undergraduate nursing curricula need further reform. The results from this study demonstrate that ongoing racial oppression is present within the undergraduate nursing curriculum, in its learning objectives. This runs counter to the aims of curriculum reform to develop a workforce that is ‘free from racism’; instead, student nurses are being taught in courses with learning objectives that racially marginalise, paternalize, and dehumanise Aboriginal and Torres Strait Islander peoples. 

There is little evidence to understand the impact of anti-racist curriculum reforms which reinforces the need for further consideration of institutional accountability measures. In light of a history where Aboriginal and Torres Strait Islander voices have been silenced in curriculum development, accreditation and monitoring, this study suggests a way forward using the RSAT in ongoing reviews of learning objectives. A range of areas were identified to target in curriculum review and reform to address the presence of racist ideals and advocate for a health care system free of racism. Further attention needs to be given as to what this means in terms of the support needed for both Indigenous and non-Indigenous academics developing curriculum that is anti-racist, ensuring that the impact of curriculum reform is monitored through institutional accountability. Understanding how racism operates even through the wording of ‘strengths-based’ and ‘culturally safe’ learning objectives is critical before we can begin to talk about what a health system ‘free from racism’ looks like.

## Figures and Tables

**Table 1:** RSAT audit of discrete Aboriginal and Torres Strait Islander peoples health courses in undergraduate nursing programs at Australian universities.

RSAT questions	*n*	%
Who wrote the learning objective?		
Aboriginal or Torres Strait Islander author	0	0%
Non-Aboriginal or Torres Strait Islander author	0	0%
Not Stated	165	100%
Is there avoidance of identifying with Aboriginal and Torres Strait Islander peoples and/or the racial experiences of Aboriginal and Torres Strait Islander peoples?		
Yes	79	47.9%
No	61	37%
N/A there is no identification of racial group either directly or indirectly	25	15.2%
Who is placed as inferior and superior in the social construction of ‘race’ within the learning objective?		
Non-Indigenous, placed as inferior	8	4.8%
Aboriginal and Torres Strait Islander peoples, placed as inferior	132	80%
N/A*—*there is no identification of racial group either directly or indirectly	25	15.2%
Is inequity explained by reference to any number of alternative factors rather than being attributable to a legacy of racism by those who belong to whiteness?		
Yes	42	25.5%
No	48	29.1%
N/A*—*where inequity is not explained either directly or indirectly	75	45.5%
Who wins and who loses based on the priorities in the learning objective?		
Upholds notions of white superiority and dominance	67	40.6%
Narratives of false neutrality	93	56.4%
Acknowledges Aboriginal and Torres Strait Islander peoples sovereignty	5	3%
What will the likely effects of the learning objective be?		
Oppressive segregation of Aboriginal and Torres Strait Islander peoples	135	83%
Towards anti-racist curriculum	9	5.5%
N/A—Learning objective is not applicable in questions 2–5	19	11.5%

**Table 2:** Emerging themes as the attributed reason for inequity in the learning objectives (n = 91), thematically analysed.

Themes	*n*
Aboriginal and Torres Strait Islander peoples (such as ‘cultural issues’ or the problematisation of ‘traditional aspects of life’)	35
Colonisation:	
Explicitly stated	6
Not-explicitly stated	19
Student personal values, beliefs, or attitudes	10
Social determinants of health	7
Communication barriers	5
Racism	4
Whiteness and/or white privilege	3
Social injustice	2

## Data Availability

The data presented in this study are available on request from the corresponding author.

## Supplementary Materials

The following supporting information can be downloaded at: https://www.mdpi.com/article/10.3390/ijerph191811455/s1, File S1: Racial Segregation Audit Tool [91].
