# Supplementary material for: Are We Teaching Nurses to Be Racist towards Aboriginal and Torres Strait Islander Peoples? A Critical Race Document Analysis of Discrete Aboriginal and Torres Strait Islander Health Courses
Source: Int J Environ Res Public Health. 2022 Sep 12;19(18):11455. doi: 10.3390/ijerph191811455 (PMC9517025; doi:10.3390/ijerph191811455)
Supplement: Supplementary file 1 [file ijerph-19-11455-s001.zip › ijerph-1882494-supplementary.pdf]

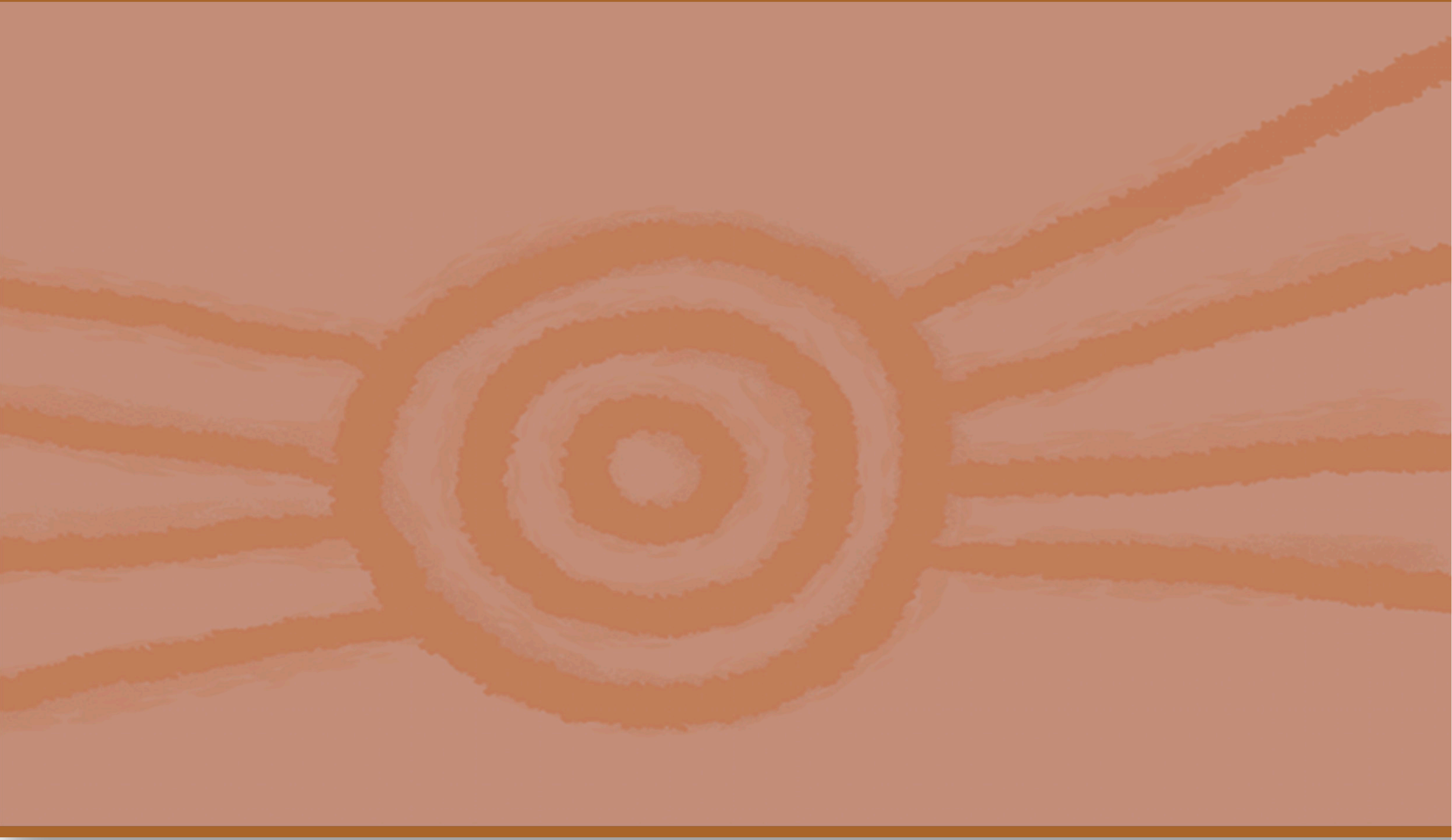

RACIAL SEGREGATION AUDIT TOOL (RSAT)

For use in evaluating health curriculum learning objectives in discrete Aboriginal and Torres Strait Islander health courses

Explanation for the Audit Tool questions

The Racial Segregation Audit Tool (RSAT) is used to evaluate health curriculum learning objectives in discrete Aboriginal and Torres Strait Islander health courses in undergraduate nursing programs. The RSAT aims to identify racism embedded within learning objectives within these courses. While racism is complex and the ways it can occur are innumerable and continuously evolving, this tool is targeted at specifically identifying where 'race' is used as a tool to oppressively segregate Aboriginal and Torres Strait Islander peoples; through both constructing and upholding racially based social hierarchies in the curriculum that prepares health professionals for entry to practice.

The RSAT focuses on student learning objectives that are used to describe the expected learning outcomes of students who are undertaking the course, as well as forming the basis for student assessment (Department of Health (DoH) 2014). In this way, the idea of reviewing the learning objectives with the RSAT, is to positively influence assessments and content within the course.

The RSAT has a series of 6 questions that draw on the works of culturally responsive pedagogies in Australia by Daniels-Mayes (2016), educational policy by Gillborn (2005) and whiteness by Leonardo (2002). The questions within the Audit Tool have been selected and guided by key documents such as the Australian Health Practitioner Regulation Agency (AHPRA) *National Scheme's Aboriginal and Torres Strait Islander Health and Cultural Safety Strategy 2020-2025* (Phillips et al. 2020) as shown in Appendix 1.

The expectation of the RSAT is that it will assist educators in critiquing university health course learning objectives, whilst also contributing to the reduction of racism within health as highlighted in nation-wide strategies for curriculum reform. The tool is structured to critique each learning objective individually and in order. Upon completion of questions 1-5, question 6 can be answered by reflecting on the preceding answers to questions 1-5. After reviewing each learning objective individually, the answers can be used to evaluate the learning objectives collectively for the course. The evaluation process will help to guide where changes and modifications may need to occur within the learning objective. The tool is readily adaptable to other health related contexts outside of nursing.

Key terms

Course: subject or unit of study which refer to components that make up a program of study

Cultural Safety: 'is an outcome of nursing education that enables safe service to be defined by those that receive the service and is achieved when the recipients of care deem the care to be meeting their cultural needs' (Ramsden 2002, p.117)

Deficit Discourse: is a racist method of thinking that frames Aboriginal and Torres Strait Islander peoples into an inferiority narrative of failure and deficiency. It is used as a means to enforce race-based inequality.

Learning objectives: also known as learning outcomes, form the basis of the unit of study (course), and guide the curriculum design and development within programs (degrees) such as a Bachelor of Nursing; they describe what students are expected to understand and learn from the course and provide the basis for the development of student assessments.

Othering: is the process of normalising whiteness and racially separating those who do not belong to whiteness.

Program: refers to the combination of courses that comprise the total award degree such as Bachelor of Nursing

Racism: is the belief that one race has the right to dominance through a narrative of superiority over all other races.

Whiteness: is a racial discourse that centre the beliefs, practices and assumptions of white people into positions of dominance and superiority, it is not the same as white people.

Question 1: Who wrote the learning objective?

This positions the authorship of the learning objective to identify potential bias as per the (O'Leary 2010, p.262) document analysis framework. More importantly however the question of authorship also indicates ownership of the narrative of Aboriginal and Torres Strait Islander peoples. This is particularly relevant where the Australian Health Practitioner Regulation Agency (Phillips et al. 2020) have defined cultural safety as determined by Aboriginal and Torres Strait Islander peoples, directly in accordance is the Australian Nursing and Midwifery Accredition Council (ANMAC) (2019) *Registered Nurse Accreditation Standard 3: Program of Study*. '*Who wrote the learning objective*' questions who holds the position of power, who is *leading* the development of learning objectives about Aboriginal and Torres Strait Islander peoples; whether it will lead to student education that will facilitate culturally safe experiences for Aboriginal and Torres Strait Isander peoples. Is authorship of the learning objective clearly stated and is the narrative driven by a person who is Aboriginal or Torres Strait Islander?

Choose either:
1. Yes
2. No
3. Not stated

Write 'Not stated' where it is not explicitly stated that the learning objectives were authored by Aboriginal or Torres Strait Islander peoples.

Question 2: Is there avoidance of identifying with a racial experience or group?

This question aims to identify whether whiteness is normalised within the learning objective; and if there is avoidance in identifying with the racial experiences of Aboriginal and Torres Strait Islander peoples. Whiteness draws power from 'othering', this normalises whiteness and racially separates those who do not belong to whiteness (Daniels-Mayes 2016; Gillborn 2005). By racially othering the experiences of Aboriginal and Torres Strait Islander peoples there can be a tendency to blame groups with an 'us and them' narrative, rather than identifying the racialised barriers imposed by whiteness (Laccos-Barrett & Brown 2021).

Example

Learning objective: 'Identify historical and cultural factors which contribute to the context of Indigenous health care problems'

Discussion: This learning objective avoids identifying with a racial experience or group by separating the racial experiences of Indigenous peoples from normative whiteness. This learning objective speaks to historical and cultural factors as causative of health inequities and these causative factors are separated from normalised whiteness by being racialised. That is, the health issues are 'Indigenous health care problems' that are separated from *non-Indigenous* health care problems.

Answer: *Yes,* there is avoidance of identifying with a racial experience or group

Choose either:
1. Yes
2. No
3. N/A

Choose N/A where there is no identification of racial group either directly or indirectly.

Question 3: Who is placed as inferior and superior in the social construction of 'race' within the learning objective?

Race is employed as a tool to justify and enable power of one group over another, to reinforce white superiority and Aboriginal and Torres Strait Islander inferiority (Daniels-Mayes 2016). Educational systems continue to pathologise Aboriginal peoples through the continued use of deficit discourse, this maintains an inferiority narrative that is rooted in innumerable raciologies (Daniels-Mayes 2016). Where question 2 aims to identify the naturalisation of whiteness in the learning objective, question 3 aims to identify if the narrative of inferiority is maintained. This can be seen in a multitude of ways, for example through the narrative of deficit either cultural, moral or biological (Daniels-Mayes 2016).

Example

Learning objective: 'Accurately describe Aboriginal and Torres Strait Islander health issues in contrast to broader population health outcomes'

Discussion: This learning objective identifies health inequities as being a part of a racialised group (inherent part of 'race'). For instance, the learning objective has a different presentation of *health issue* for Aboriginal and Torres Strait Islander people compared to *outcome* for those who are not Aboriginal or Torres Strait Islander; as well as problematising Aboriginal and Torres Strait Islander peoples, this learning objective upholds the narrative of inferiority of Aboriginal and Torres Strait Islander peoples compared to 'the broader population'.

Answer: *Indigenous, placed as inferior*

Choose either:

1. Non-Indigenous, placed as inferior
2. Aboriginal and Torres Strait Islander peoples, placed as inferior
3. N/A

Choose N/A where there is no identification of racial group either directly or indirectly.

Question 4: Is inequity explained by reference to any number of alternative factors rather than being attributable to a legacy of racism by those who belong to whiteness?

The minimisation of a racist legacies, including colonisation and dispossession is a defining characteristic of whiteness (Leonardo 2000). This question seeks to determine whether racism (e.g., colonisation), in all its myriads, is or is not identified as the cause of inequity. This can be seen through racialisation in many ways that blame the groups who do not belong to whiteness (Laccos-Barrett & Brown 2021). i.e., health inequalities due to cultural differences or language barriers rather than the ongoing racist legacy of colonisation and whiteness as the primary source of inequity.

Example

Learning Objective: 'Evaluate how social and cultural factors shape the health beliefs, experiences, and outcomes of Aboriginal and Torres Strait Islanders and other cultural groups'

Discussion: This learning objective attributes health inequities to social and cultural factors of Aboriginal and Torres Strait Islander peoples. The objective blames inequities on the differences as

comparable to normative whiteness, yet inequitable health outcomes are not attributed to racism by those who belong to whiteness.

Answer: *Yes*, the learning objective blames cultures of racialised groups 'other' rather than factors attributable to the legacy whiteness as the basis for inequity.

Choose either:
1. Yes (explain)
2. No
3. Choose N/A where inequity is not explained either directly or indirectly.

Question 5: Who wins and who loses based on the priorities in the learning objective?

Does the learning objective seek to foreground Aboriginal and Torres Strait Islander peoples sovereignty, or does it maintain notions of white superiority and dominance through educating health professionals towards racist behaviours such as paternalism, prejudice, low expectations and white saviorism? Aboriginal and Torres Strait Islander peoples should talk towards histories of resistance and struggle, emancipation and success; sovereignty and self-determination (Moodie 2018). Specifically does the learning objective foreground Aboriginal and Torres Strait Islander peoples sovereignty. Questioning whether Aboriginal peoples are positioned as experts in their own health and whether the effect of the learning objective asserts this. Where a learning objective refuses to acknowledge Aboriginal and Torres Strait Islander peoples sovereignty, it is prudent to question who the learning objective seeks to serve, the outcome of which is explored is question 6.

Example

Learning Objective: 'Become familiar with Aboriginal and Torres Strait Islander peoples holistic and traditional health practices and appreciate their incorporation into a western framework of health'.

Discussion: A learning objective such as this talks to assimilation of Aboriginal and Torres Strait Islander peoples health into a western framework, this reinforces white superiority by placing the western framework as the 'norm'. This learning objective maintains white superiority and dominance through upholding the narrative of Aboriginal and Torres Strait Islander inferiority. Additionally, the learning objective speaks *appreciation* of the incorporation of Aboriginal and Torres Strait Islander health beliefs and practices into a western framework rather than acknowledging Aboriginal and Torres Strait Islander peoples sovereign beliefs and practices outside of white dominance.

Answer: This learning objective *maintains white dominance and superiority* by not centering the sovereignty of Aboriginal and Torres Strait Islander peoples and knowledge and by further working towards assimilation into a perceived superior westernised model of health.

An example of 'perceived neutrality' would be in a learning objective where whiteness is not positioned either directly or indirectly into a position of superiority, but where Aboriginal and Torres Strait Islander peoples sovereignty is not acknowledged. In such considerations it is imperative to recognise that by default whiteness is positioned in place of inherent superiority.

Choose either:
1. Upholds notions of white superiority and dominance

2. Narratives of false neutrality
3. Acknowledges Aboriginal and Torres Strait Islander peoples sovereignty

Question 6: What will the likely effects of the learning objective be?

The outcome of the learning objective in a health context, is it towards healthcare that can be perceived as culturally safe and 'free of racism' or does it perpetuate oppressive segregation. This question considers the cumulative answers of questions 1-5. Based on the answers, do you consider that the learning objective facilitates the education of the health professional towards care that can be perceived as culturally safe and free of racism? Or does the learning objective assert the social construction of race as a tool of oppressive segregation?

> 'Cultural safety is determined by Aboriginal and Torres Strait Islander individuals, families and communities. Culturally safe practise is the ongoing critical reflection of health practitioner knowledge, skills, attitudes, practising behaviours and power differentials in delivering safe, accessible and responsive healthcare free of racism.'
>
> Australian Health Practitioner Regulation Agency (Phillips et al. 2020)

Choose either:
1. Oppressive segregation of Aboriginal and Torres Strait Islander peoples
2. Towards anti-racist curriculum
3. N/A- The learning objective is not applicable to questions 2-5.

Appendix 1. Mapping the Audit Tool to significant documents

University<br>/Course	Learning Objective	1. Who wrote the learning objective?	2. Is there avoidance of identifying with a racial experience or group?	3. Who is placed as inferior and superior in the social construction of 'race' within the learning objective?	4. Is inequity explained by reference to any number of alternative factors rather than being attributable to dispossession and colonisation by those who belong to whiteness?	5. Who wins and who loses based on the priorities in the learning objective?	6. What will the effects of the learning objective be?
ANMAC RN Accreditation Standards 2019<br>registerednurseaccreditationstandards2019_0.pdf (anmac.org.au)	Standard 3: Program of study<br>3.5 The program's content and subject learning outcomes ensure: a. achievement of the NMBA Registered nurse standards for practice (9)	Standard 2: Governance<br>2.3 The education provider undertakes consultation into the design and ongoing management of the program from external representatives of the nursing profession, including Aboriginal and/or Torres Strait Islander peoples, consumers,				Standard 3: Program of study<br>3.7 The program's content and subject learning outcomes embed principles of diversity, culture, inclusion and cultural safety for all people.	Standard 1: Safety of the public<br>1.1 The program's guiding principle is safety of the public.<br>1.2 The program is delivered in Australia to prepare graduates for safe and ethical practice.

		students, carers and other relevant stakeholders.					
NMBA Nursing and Midwifery Board of Australia - Registered nurse standards for practice (nursingmidwifery board.gov.au)			Standard 3: Maintains the capability for practice<br>3.3 uses a lifelong learning approach for continuing professional development of self and other	Standard 1: Thinks critically and analyses nursing practice<br>1.3 respects all cultures and experiences, which includes responding to the role of family and community that underpin the health of Aboriginal and Torres Strait Islander peoples and people of other cultures	Standard 1: Thinks critically and analyses nursing practice<br>1.2 develops practice through reflection on experiences, knowledge, actions, feelings and beliefs to identify how these shape practice	Standard 2: Engages in therapeutic and professional relationships<br>2.3 recognises that people are the experts in the experience of their life	Standard 2: Engages in therapeutic and professional relationships<br>2.2 communicates effectively, and is respectful of a person's dignity, culture, values, beliefs and rights<br><br>2.5 advocates on behalf of people in a manner that respects the person's autonomy and legal capacity<br><br>2.7 actively fosters a culture of safety and learning that includes engaging with health professionals and others, to share knowledge and practice that supports person-centred care

							Standard 4: Comprehensively conducts assessments<br>4.1 conducts assessments that are holistic as well as culturally appropriate
AHPRA<br>Aboriginal and Torres Strait Islander Health and Cultural Safety Strategy 2020-2025		Definition: Cultural safety is determined by Aboriginal and Torres Strait Islander individuals, families and communities. Culturally safe practise is the ongoing critical reflection of health practitioner knowledge, skills, attitudes, practising behaviours and power differentials in delivering safe, accessible and responsive healthcare free of racism.	To ensure culturally safe and respectful practice, health practitioners must:<br>b. Acknowledge and address individual racism, their own biases, assumptions, stereotypes and prejudices and provide care that is holistic, free of bias and racism;	To ensure culturally safe and respectful practice, health practitioners must:<br>b. Acknowledge and address individual racism, their own biases, assumptions, stereotypes and prejudices and provide care that is holistic, free of bias and racism;	To ensure culturally safe and respectful practice, health practitioners must:<br>a. Acknowledge colonisation and systemic racism, social, cultural, behavioural and economic factors which impact individual and community health;	To ensure culturally safe and respectful practice, health practitioners must:<br>c. Recognise the importance of self-determined decision-making, partnership and collaboration in healthcare which is driven by the individual, family and community;	To ensure culturally safe and respectful practice, health practitioners must:<br>d. Foster a safe working environment through leadership to support the rights and dignity of Aboriginal and Torres Strait Islander people and colleagues
UN United Nations Declaration on the Rights of Indigenous Peoples	Article 15<br>1. Indigenous peoples have the right to the dignity and diversity of their cultures, traditions, histories and	Article 18<br>Indigenous peoples have the right to participate in decision-making in matters which would affect their rights,				Article 29<br>3. States shall also take effective measures to ensure, as needed, that programmes for monitoring,	Article 21<br>1. Indigenous peoples have the right, without discrimination, to the improvement of their economic and

	aspirations which shall be appropriately reflected in education and public information.	through representatives chosen by themselves in accordance with their own procedures, as well as to maintain and develop their own indigenous decision-making institutions.				maintaining and restoring the health of indigenous peoples, as developed and implemented by the peoples affected by such materials, are duly implemented	social conditions, including, inter alia, in the areas of education, employment, vocational training and retraining, housing, sanitation, health and social security.<br><br>Article 24<br>2. Indigenous individuals have an equal right to the enjoyment of the highest attainable standard of physical and mental health. States shall take the necessary steps with a view to achieving progressively the full realization of this right.
NMBA Code of Conduct for Nurses			Principle 3: Cultural practice and respectful relationships<br><br>3.2 Culturally safe and respectful practice<br><br>Culturally safe and respectful practice	Principle 3: Cultural practice and respectful relationships<br><br>3.2 Culturally safe and respectful practice<br><br>Culturally safe and respectful practice	Principle 3: Cultural practice and respectful relationships<br>3.1 Aboriginal and/or Torres Strait Islander peoples' health Understanding and acknowledging historic factors such as colonisation and	Principle 3: Cultural practice and respectful relationships<br><br>3.2 Culturally safe and respectful practice<br><br>Culturally safe and respectful practice	Principle 3: Cultural practice and respectful relationships<br>3.1 Aboriginal and/or Torres Strait Islander peoples' health<br>a. Provide care that is holistic, free of bias and racism, challenges belief

			requires having knowledge of how a nurse’s own culture, values, attitudes, assumptions and beliefs influence their interactions with people and families, the community and colleagues.	requires having knowledge of how a nurse’s own culture, values, attitudes, assumptions and beliefs influence their interactions with people and families, the community and colleagues.	its impact on Aboriginal and/or Torres Strait Islander peoples’ health helps inform care.<br><br>c. Acknowledge the social, economic, cultural, historic and behavioural factors influencing health, both at the individual, community and population levels	requires having knowledge of how a nurse’s own culture, values, attitudes, assumptions and beliefs influence their interactions with people and families, the community and colleagues.	based upon assumption and is culturally safe and respectful for Aboriginal and/or Torres Strait Islander peoples<br>b. Advocate for and act to facilitate access to quality and culturally safe health services for Aboriginal and/or Torres Strait Islander peoples, and<br>c. Recognise the importance of family, community, partnership and collaboration in the healthcare decision-making of Aboriginal and/or Torres Strait Islander peoples, for both prevention strategies and care delivery.

Australian Nursing and Midwifery Accredition Council (ANMAC) 2019, *Registered Nurse Accreditation Standards*, Australian Nursing and Midwifery Council, viewed 25th March 2020, <https://www.anmac.org.au/sites/default/files/documents/registerednurseaccreditationstandards2019.pdf>.

Daniels-Mayes, SM 2016, 'Culturally responsive pedagogies of success: improving educational outcomes for Australian Aboriginal students', School of Communication, International Studies and Languages, University of South Australia. viewed 11th of February 2021, <https://find.library.unisa.edu.au/primo-explore/fulldisplay?vid=UNISA&search_scope=All_Resources&docid=UNISA_ALMA11145960000001831>.

Department of Health (DoH) 2014, *Aboriginal and Torres Strait Islander Health Curriculum Framework*, Commonwealth of Australia, Canberra, viewed 14th of June 2020, <https://www1.health.gov.au/internet/main/publishing.nsf/Content/72C7E23E1BD5E9CFCA257F640082CD48/$File/Health%20Curriculum%20Framework.pdf>.

Gillborn, D 2005, 'Education policy as an act of white supremacy: whiteness, critical race theory and education reform', *Journal of education policy,* vol. 20, no. 4, pp. 485-506.

Laccos-Barrett, K & Brown, A 2021, 'Policy and advocacy in culturally diverse health care', in T Dune, K McLeod & R Williams (eds), *Culture, diversity and health in Australia: Towards culturally safe health care*, Routledge New York, pp. 74-91.

Leonardo, Z 2002, 'The Souls of White Folk: Critical pedagogy, whiteness studies, and globalization discourse', *Race, ethnicity and education,* vol. 5, no. 1, pp. 29-50.

Lorde, A 1992, 'Age, race, class and sex: Women redefining difference', in M Anderson & P Hill Collins (eds), *Race, class and gender: An anthology* Wadsworth Belmont, CA, pp. 495-502.

Moodie, N 2018, 'Decolonising race theory: Place, survivance and sovereignty', in G Vass et al (eds), *The Relationality of Race in Education Research*, 1 edn, Routledge, London, pp. 33-46.

O'Leary, Z 2010, *The essential guide to doing your research project*, Sage Publications, Thousand Oaks, CA.

Phillips, G, Brayshaw, J, Fletcher, M & Callister, G 2020, *National Scheme's Aboriginal and Torres Strait Islander Health and Cultural Safety Strategy 2020-2025*, Australian Health Practitioner Regulation Agency, Canberra, viewed 16th January 2021, <https://www.ahpra.gov.au/About-AHPRA/Aboriginal-and-Torres-Strait-Islander-Health-Strategy/Aboriginal-and-Torres-Strait-Islander-Health-Strategy-Group.aspx>.

Ramsden, I 2002, 'Cultural safety and nursing education in Aotearoa and Te Waipounamu', Doctoral Dissertation thesis, Victoria University of Wellington. <https://www.croakey.org/wp-content/uploads/2017/08/RAMSDEN-I-Cultural-Safety_Full.pdf>.
